# Bacteremic patients in the Emergency Department – how do they present and what is the diagnostic validity of temperature, CRP and SIRS?

**DOI:** 10.1186/1757-7241-21-S2-A19

**Published:** 2013-09-09

**Authors:** Katrine Prier Lindvig, Stig Lønberg Nielsen, Daniel Henriksen, Thøger Gorm Jensen, Hans Jørn Kolmos, Court Pedersen, Annmarie Touborg Lassen

**Affiliations:** 1 Department of Emergency Medicine Odense University Hospital, Denmark; 2 Department of Infectious Diseases Odense University Hospital, Denmark; 3 Department of Clinical Microbiology Odense University Hospital, Denmark

## Background

It might be a clinical challenge to identify patients with bacteremia. Blood cultures are often ordered based on the symptoms of fever and chills. Detailed knowledge of the clinical presentation of acute medical patients will improve the identification of bacteremic patients. The aim of this study was to evaluate the diagnostic value of temperature (°C), C-reactive-protein (CRP), and Systemic Inflammatory Response Syndrome (SIRS) in bacteremic patients admitted to the Medical Emergency Department (ED).

## Methods

A population based cohort study including all adult (>15 years old) first-time admissions at the ED at Odense University Hospital between 1/8 2009-31/8 2011. A bacteremic patient was defined as having a positive blood culture drawn within the first two days after admission. All patients had their bloodpressure, pulse rate, respiratory frequency, oxygen saturation, level of consciousness measured and standard blood samples drawn at arrival.

## Results

We included 11.996 acute medical patients and excluded31 patients because of missing identification data. Median age was 66 years (range 15-103), and 5499 (45.0%) were male. In total 5503 (45.9%) patients had blood cultures performed, of which 418 (7.6%) were culture positive, defining bacteremia. Of the 418 bacteremic patients, 381 had a temperature measured at arrival; hereof 130 (34.1%) patients had a normal rectal temperature (36.0°-38.0°C) registered, 116 (28 %) had a CRP<100mg/dL, and 102 (24%) did not fulfil the criteria for SIRS. The most frequent species among the 130 patients with normal temperature were E.coli n=39 (30%), S.aureus n=19 (15%) and S.pneumoniae n=13 (10%). The ROC-area for CRP and temperature as predictors of bacteraemia were 0.67 and 0.75 respectively, representing a sensitivity of 0.66 and a specificity of 0.82 with a CPR-cut-off-value of 100, and a sensitivity of 0.59 and a specificity of 0.84 with a temperature-cut-off-value of ≥38.0°C.

## Conclusion

34% of the acute medical bacteremic patients had a normal temperature when arriving at the hospital, 32% had a CRP below 100 mg/dL and 24% did not fulfil the criteria for SIRS. If the decision to order blood cultures were based on either temperature, CRP or SIRS, one third of all bacteremic patients would have been overlooked.

